# Women's preferences for a new contraceptive under development: an exploratory study

**DOI:** 10.3389/fgwh.2023.1095112

**Published:** 2023-07-21

**Authors:** Tessa Madden, Sarah Y. Cohen, Rachel Paul, Emily G. Hurley, Michael A. Thomas, Giovanni Pauletti

**Affiliations:** ^1^ Divisions of Family Planning & Clinical Research, Department of Obstetrics and Gynecology, Washington University in St. Louis School of Medicine, St. Louis, MO, United States; ^2^ Division of Reproductive Endocrinology and Infertility, Department of Obstetrics and Gynecology, University of Cincinnati College of Medicine, Cincinnati, OH, United States; ^3^ Department of Pharmaceutical and Administrative Sciences, University of Health Sciences and Pharmacy in St. Louis, St. Louis College of Pharmacy, St. Louis, MO, United States

**Keywords:** contraception, contraceptive preferences, contraceptive development, MTurk, nonhormonal

## Abstract

**Objective:**

Currently available contraceptive methods do not meet the needs of all users. We sought to explore preferences of potential end-users regarding an on-demand, non-hormonal female contraceptive currently under development, using a web-based survey.

**Study design:**

We recruited respondents for an exploratory survey via web link on Amazon Mechanical Turk (MTurk). Individuals were eligible if they were 18–44 years of age, identified as cis-gender female, were English-speaking, not pregnant, and had used barrier contraception previously. Respondents provided demographic characteristics and a basic reproductive history. We then provided a brief description of the potential contraceptive. Respondents were asked about their interest in the proposed contraceptive and preferences for method attributes.

**Results:**

A total of 500 respondents completed the survey. Three-quarters of respondents were <35 years of age and 48.2% were currently using a barrier contraceptive method. Three-fourths of respondents (73.8%) expressed interest in using the contraceptive under development. The majority wanted the method to be small (≤2 inches), rod-shaped, and low cost (<$5 per use). More than half (59.4%) said it was important to be able to use the method without partners’ knowledge. The most reported potential concerns were vaginal irritation (51.6%) and lack of effectiveness (46.4%). Sixty percent of respondents were confident they could use the method correctly.

**Discussion:**

Available contraceptive methods lack attributes preferred by some users. Development of new contraceptives frequently does not involve end-user input early in the development process. Individuals in this sample displayed interest in the proposed contraceptive and expressed preferences that can inform the further development of this method.

## 1. Introduction

Contraception is a cornerstone of reproductive health. However, approximately 30% of reproductive-age US women who do not desire pregnancy were not using contraception at their last sexual encounter (1). There are several coitally-dependent, or on-demand, non-hormonal methods available in the United States including male and female condoms, diaphragms and cervical caps, spermicides, and contraceptive gels (2). While 12.9% of contraceptive users in the United States rely on male condoms as their primary contraceptive method, the proportion that relies on other coitally-dependent methods is less than 1% (3). Male condoms may be used in combination with another method: approximately one-quarter of reproductive-age women reported using a male condom during their most recent sexual intercourse (4).

Not all available on-demand contraceptives possess the attributes users deem most important, including effectiveness, low risk of side effects, affordability, ease of access and use, and female-controlled (5). Prior studies have reported one-third of women feel that contraceptive use only at the time of sex is “extremely important” and more than two-thirds of women prefer to determine if and when contraception is used (5, 6). While widely used, male condoms do not have all the attributes many individuals value in a contraceptive method, such as being user-controlled. On-demand, female-controlled methods, such as female condoms and spermicides also have barriers to use; patients frequently report female condoms are difficult or uncomfortable to insert, interfere with sexual experience, and partners object to the method (7, 8). Moreover, more than 50% of women prefer methods that can be concealed from a partner (5, 6). Currently available methods that are more difficult for a partner to detect, such as spermicides, can increase the risk of vaginal irritation, allergic reactions, and sexually transmitted infection (STI) acquisition (2).

The gap in available contraception with preferred attributes may be filled with new contraceptive technologies, and input from end users regarding their preferences can optimize a new method development. We sought to explore the preferences of potential end users regarding an on-demand, non-hormonal method of contraception currently under development.

## 2. Materials and methods

We recruited a convenience sample of respondents through Amazon Mechanical Turk (MTurk). MTurk is a web service frequently used for research recruitment in the social sciences (9). Compared to other in-person convenience sampling tools, MTurk has been shown to generate a sample more representative of the general U.S. population (10). The platform allows each worker to complete a task only once, ensuring unique respondents. MTurk workers review a list of available tasks and select tasks to complete. Worker who selected our task clicked a link that redirected them to the survey in Research Electronic Data Capture tools (REDCap) hosted at Washington University (11, 12). REDCap is a secure, HIPAA compliant, web-based application. We did not collect any protected health information from respondents.

Individuals were eligible to participate if they lived in the United States, were of reproductive age (18–44 years) English-speaking, identified as a cis-gender female, were not pregnant, and had previously used barrier contraception. At the halfway point of recruitment (*n* = 250 enrolled), we limited enrollment to individuals currently using a barrier method and excluded individuals who had undergone surgical sterilization (tubal ligation or hysterectomy) to increase the sample of individuals who might be potential users of the proposed contraceptive. Eligible individuals reviewed a consent information sheet prior to beginning the survey. They were given the option to exit the browser window if they did not wish to participate. The project was approved by the Washington University in St. Louis School of Medicine Institutional Review Board prior to recruitment.

Eligible individuals completed the survey through a web link. Survey responses were verified by including random attention test questions alongside survey questions. Respondents who completed the survey with accurate attention test responses were compensated with a $2.14 deposit into their Amazon Payments account determined based on minimum wage calculations relative to survey length and time to completion (13).

The survey began with screening questions to determine eligibility, followed by questions to gather respondent characteristics and a brief reproductive history. We asked respondents about the primary reason for prior or current use of male and/or female condoms (if applicable), STI history, and whether they had ever felt pressured by their partner to not use contraception to prevent pregnancy or STIs.

**Figure 1:**
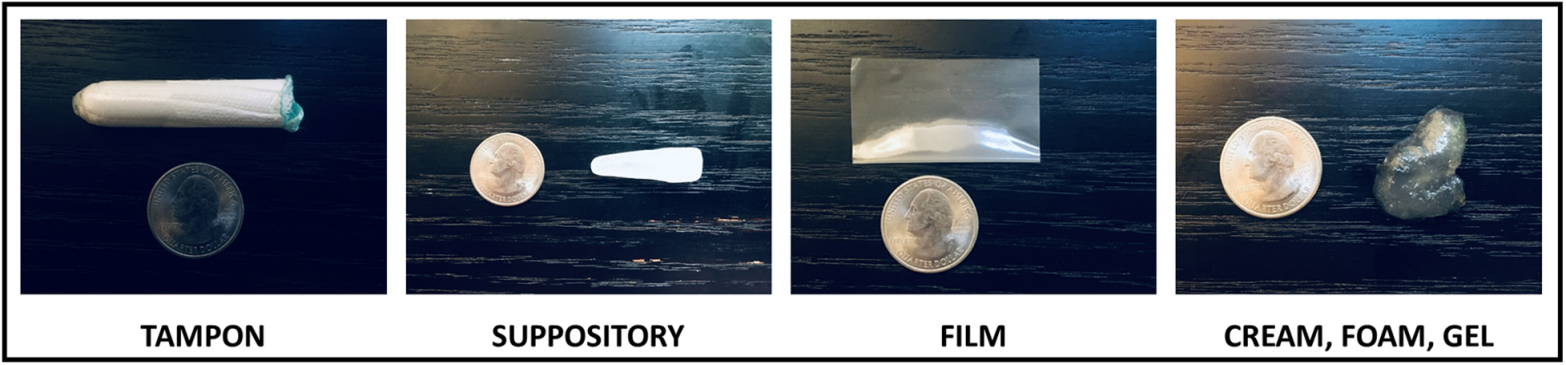
Photos presented to participants for their input on shape of contraceptive method.

Following these questions, we showed respondents the following prompt introducing the novel contraceptive method currently in development, “*A new type of birth control is being designed. You would put it in the vagina before having sex. The birth control would start as a fabric-like, delicate material and dissolve into a gel that would protect against pregnancy and sexually transmitted infections. It is a method you could use without your partner knowing.”* After reading the prompt, we asked respondents if they would be interested in using the novel contraceptive. Subsequent questions elicited feedback on product characteristics such as preferred size of the product, largest size the respondent would be comfortable with, how quickly they would expect the product to work, how long they would expect the effects to last, how much they would be willing to pay per use, the importance of using the method without the partner knowing, where they would expect to be able to purchase the product, and concerns they had about using it. We showed respondents photos of a tampon, suppository, contraceptive film, and spermicide next to a quarter coin for scale (Figure 1) and asked how much they would like a new method in the different shapes on a 5-point Likert scale from 1 (strongly dislike) to 5 (strongly like).

We performed statistical analysis using SPSS, Version 27 (IBM Corp., Armonk, NY). We used descriptive statistics to characterize the respondents as well as their responses regarding features and characteristics of the proposed new contraceptive method and potential concerns and confidence about using the proposed new method.

## 3. Results

We administered the surveys from August 2020 to September 2020. A total of 3,406 individuals opened the survey; of these 825 were eligible (24.2%). Of the eligible individuals, 200 did not complete the survey and 125 failed the attention checks, leaving a final sample of 500 individuals (60.6%). Respondents were predominantly white (74.4%) and non-Hispanic (83.8%). The majority were ages 25–34 (69.8%). Current barrier method use was reported by 48.2%. Almost one third (29.0%) had a history of a STI and 42.0% reported a prior unintended pregnancy. Half the respondents (51.2%) reported having previously been pressured by a partner to not use contraception (Table 1).

**Table 1:** Demographic and reproductive characteristics of respondents.

Characteristic	Total
	*N* = 500
Age, median (IQR)	30 (25–34)
Hispanic	81 (16.2)
Race	
American Indian, Native American	5 (1.0)
Asian or Pacific Islander	14 (2.8)
Black, African American	97 (19.4)
White	372 (74.4)
Other, Multiracial	6 (1.2)
Did not report	6 (1.2)
Any history of barrier method use	342 (68.4)
Current barrier method use	241 (48.2)
History of unintended pregnancy	
Yes	210 (42.0)
No	290 (58.0)
History of STI	
Yes	145 (29.0)
No	327 (65.4)
Unsure	28 (5.6)
Reason for using male condom^a^	
Mostly pregnancy prevention	117 (49.6)
Mostly STI prevention	51 (21.6)
Both	68 (28.8)
Uses tampons with period	
Yes	331 (66.2)
No	169 (33.8)
Pressured by partner to not use contraception	
Yes	256 (51.2)
No	244 (48.8)
Pressured by partner to not use contraception to prevent STIs	
Yes	220 (44.0)
No	280 (56.0)

^a^ Denominator is 236 that reported ever using a male condom.

**Figure 2:**
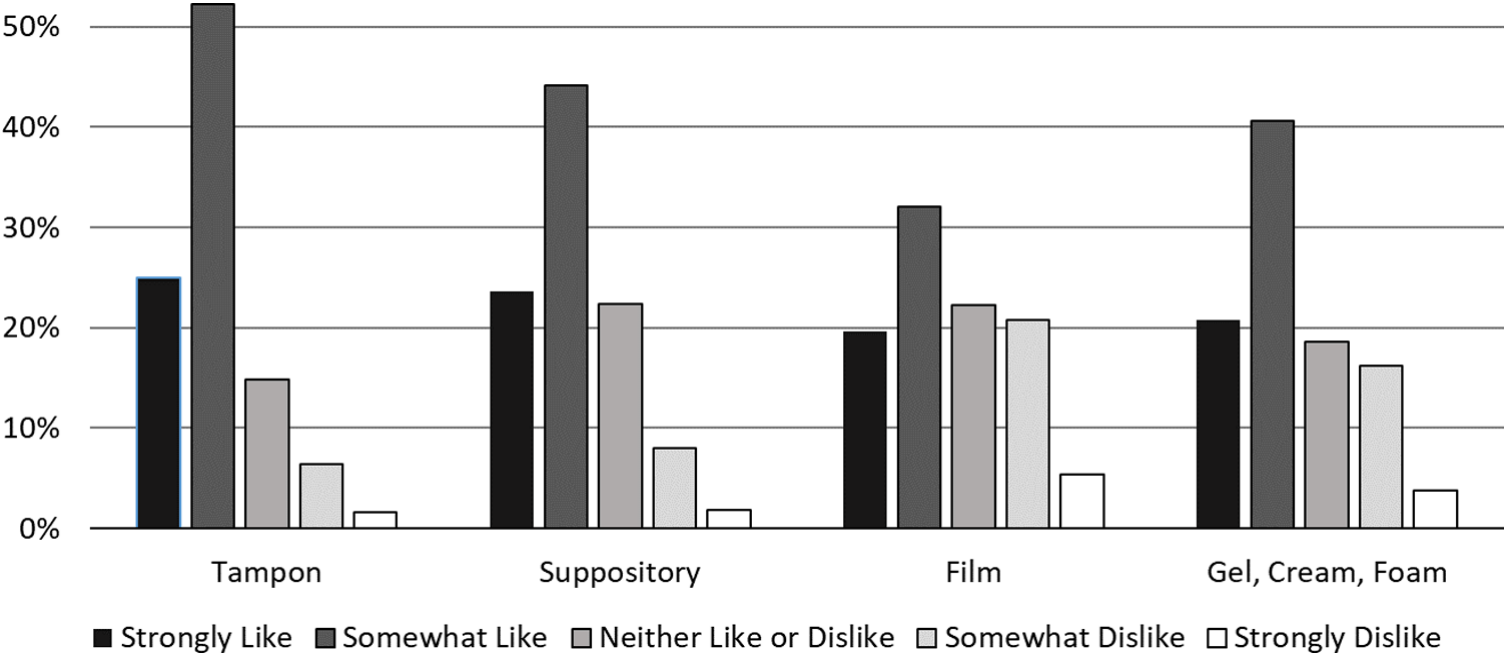
Distribution of preferences for shapes for proposed novel contraceptive.

Nearly three-quarters (73.8%) of respondents reported interest in using the proposed contraceptive while 9.6% were unsure. While preferences about contraceptive attributes varied, a method two inches or smaller, effective within five minutes, lasting at least 3–6 h, cost less than $5 per use, and could be used without a partner's knowledge was preferred by over half of the sample (Table 2). Two-thirds of respondents reported using tampons with their period and a tampon shape was the most preferred of the presented options for the new contraceptive (Figure 2). Approximately half of respondents expected that were this method available, they could obtain it at a retail location such as a drug store, supermarket, or large retail store (Table 2).

**Table 2:** Participant feedback on attributes of a proposed novel contraceptive.

Product feedback	Total
	*N* = 500
Interested in using method	
Yes	369 (73.8)
No	83 (16.6)
Unsure	48 (9.6)
Comfortable placing in vagina without applicator	
Yes	342 (68.4)
No	99 (19.8)
Unsure	59 (11.8)
Preferred product size	
Smaller than 1 inch	133 (26.6)
1–2 inches	269 (53.8)
3 inches or larger	98 (19.6)
Largest product size would use	
Smaller than 1 inch	102 (20.4)
1–2 inches	247 (49.4)
3 inches or larger	151 (30.2)
How quickly would you EXPECT the product to work?	
Immediately	77 (15.4)
Within 5 min	250 (50.0)
Within 10 min	143 (28.6)
Within 15 min	30 (6.0)
How long would you expect the effects of this product to last?	
1–2 h	120 (24.0)
3–6 h	307 (61.4)
7–12 h	51 (10.2)
More than 12 h	22 (4.4)
How much would you be willing to pay per use?	
< $1	40 (8.0)
< $3	144 (28.8)
< $5	206 (41.2)
< $10	81 (16.2)
< $15	29 (5.8)
Important to use without partner knowing	
Yes	296 (59.2)
No	172 (34.4)
Unsure	32 (6.4)
Where would you expect to be able to buy the product?	
Drug store	264 (52.8)
Grocery store or supermarket	236 (47.2)
Large retail store	226 (45.2)
Clinic or health center	190 (38.0)
Prescription from doctor	171 (34.2)
Gas station	100 (20.0)

**Figure 3:**
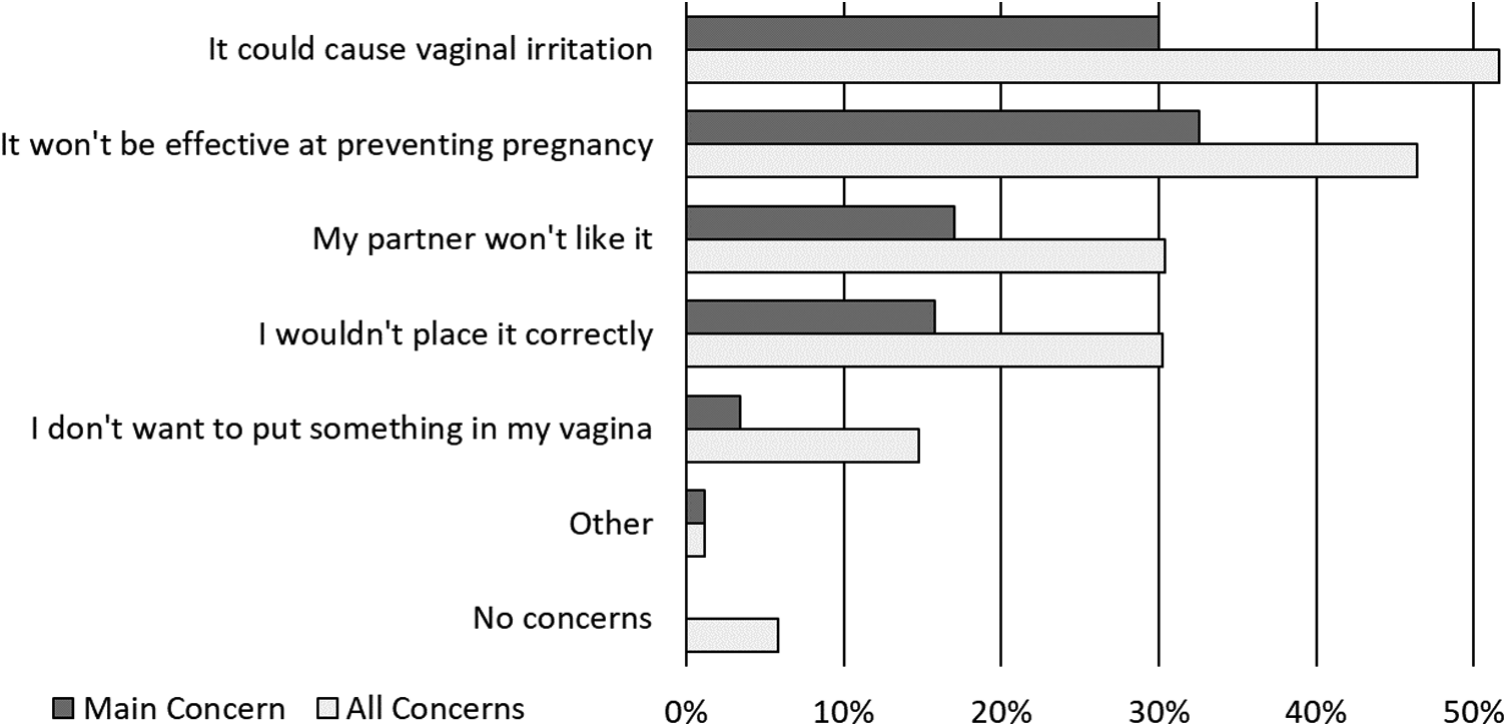
Patients concerns, and main concern, about using the novel contraceptive.

Respondent concerns about using the contraceptive were most commonly about vaginal irritation (51.6%) and lack of effectiveness at preventing pregnancy (46.4%) (Figure 3). The majority of respondents (59.8%) were confident they could use the proposed contraceptive correctly, only 2.0% of respondents were “not at all confident” they could use the contraceptive correctly.

## 4. Discussion

In this study, over two-thirds of the respondents expressed interest in using the proposed, on-demand contraceptive. When asked about potential characteristics of the method, respondents preferred the contraceptive to be similar in shape and size to a tampon and low in cost. Over half the respondents said it was important to be able use the method without their partner knowing, a significant finding given that nearly half of respondents reported prior experience with partner coercion to not use contraception. Respondents’ most common concerns about use of the proposed contraceptive were vaginal irritation and lack of effectiveness.

Side effects and effectiveness have been previously shown to be principal considerations for individuals when choosing a contraceptive method (5, 14, 15). In two studies conducted in low- and middle-income countries, health concerns, including concerns about side effects, were the most common reason for contraceptive nonuse (14, 15). In three different U.S. surveys, contraceptive effectiveness was commonly identified as “extremely important” by greater than 80% of respondents. These surveys also found that a low risk of side effects was also important to approximately 75% of respondents (5, 6, 16). Although our respondents also identified contraceptive effectiveness and potential side effects as important concerns, the overall percentages who cited these concerns were lower in our study compared to previous studies.

Prior studies have shown that partner opinion influences likelihood of contraceptive use (5, 8, 14, 15, 17, 18). A study of South African university students found that women were more likely to use female condoms if their partner had a “positive attitude” toward the condoms (17). Another international study identified partner opposition as a reason for contraceptive nonuse (15). Users may prefer independent control of contraception and methods that cannot be detected by partners. In a 2012 survey of U.S. women at risk of unintended pregnancy, 70% of respondents identified the ability to control the use of contraception as an “extremely important” contraceptive feature and an “undetectable” contraceptive method was classified as “extremely important” by 57% of respondents (5). A study of California adolescents found that about 60% of respondents preferred that their contraceptive method was “partner-independent” (16). A diverse group of respondents from six states found control over method use (71%), responsibility for method use (62%) and imperceptible method (55%) to be “extremely important.” Black and Hispanic women were more likely to rate these features as “extremely important” than their white counterparts (6). Though our findings are similar to prior studies, we found a smaller majority of respondents who preferred a method which could not be detected by their partner. Because our sample was predominantly white and non-Hispanic, this difference may be explained by racial and ethnic variations in contraceptive preferences (6).

Although some studies have assessed women's contraceptive preferences related to the attributes of existing methods, there has been limited end-user involvement in the development of new contraceptives. End-user feedback has been shown to assist in development of effective reproductive health tools, such as contraceptive decision aids (19). However, end user input into new contraceptive development, especially early in the development process, has been limited. Greater emphasis has been placed on technical feasibility rather than user preference, particularly during the development of non-hormonal methods (20, 21). Thus, the National Institutes of Health has made it a priority to include end-user input early in the contraceptive development process (22). Additionally, the FDA and international medical device regulatory authorities require the incorporation of user input into the design process (23–25). Our survey aimed to identify user preferences for this contraceptive under development with a focus on design features, such as size, shape, time to efficacy and length of efficacy which may impact willingness to use the method.

Strengths of this study included a broad sample—we enrolled respondents from across the United States via MTurk. The use of MTurk allows for broader recruitment of respondents, including outside of clinical or research settings. The MTurk platform also permitted continued recruitment throughout the COVID-19 pandemic. Respondents were sexually active, reproductive-age females at risk of STIs and unintended pregnancy, which is the segment of the population most likely to use barrier contraception. Furthermore, three-quarters of our sample was less than 35 years old, which is appropriate given that 25–34-year-old women not seeking pregnancy have the highest rate of contraceptive use and 20–39-year-old women have the highest rate of condom use compared to other age groups (26, 27).

Our study does have some limitations. Only English-speaking respondents in the United States, most of whom had used a barrier method previously, were eligible. In addition, our sample was predominantly white and non-Hispanic. Therefore, these findings may not be generalizable to other potential users. We also changed our eligibility criteria after half the respondents completed the survey to better identify respondents who could be potential end users which could further limit generalizability. The data presented relies on respondents’ self-report which is subject to self-report bias. This may be more likely for responses to sensitive questions which the survey included. We asked respondents about a product which has not yet been developed, which could result in an overestimation of the likelihood of real-world use. We also stated in the description of the proposed contraceptive it could be used without partners’ knowledge, which may have biased respondents towards identifying this as an important attribute, although our findings are similar to other studies. Several studies have raised potential limitations of the use of MTurk, particularly related to the characteristics of MTurk samples (28–30). MTurk respondents tend to be more politically liberal, have higher education levels, less religiosity, and lower life satisfaction (10). Furthermore, participation in the MTurk platform requires internet access, use of Amazon, and awareness of the platform's existence.

Our results provide further insights about user preferences for contraceptive attributes and design. Understanding the importance of a familiar shape and insertion technique could improve users’ comfort with a new method. Incorporating end users’ feedback early in the design process is critical for successful device development. Respondents in this sample indicated an interest in hypothetical product with potential to be effective at preventing pregnancy, sold at low cost, and used with few side effects. The development of this novel contraceptive could further expand individuals’ choices ultimately improving contraceptive uptake and continuation.

## Data Availability

The raw data supporting the conclusions of this article will be made available by the authors upon request, without undue reservation.
